# *Eucommia ulmoides* bark extract reduces blood pressure and inflammation by regulating the gut microbiota and enriching the *Parabacteroides* strain in high-salt diet and N(omega)-nitro-L-arginine methyl ester induced mice

**DOI:** 10.3389/fmicb.2022.967649

**Published:** 2022-08-18

**Authors:** Dong Yan, Wenhao Si, Xiaoyue Zhou, Mengjie Yang, Yuanhang Chen, Yahan Chang, Yidan Lu, Jieyu Liu, Kaiyue Wang, Moyu Yan, Feng Liu, Min Li, Xianliang Wang, Minna Wu, Zhongwei Tian, Haiyan Sun, Xiangfeng Song

**Affiliations:** ^1^Xinxiang Key Laboratory of Pathogenic Biology, Department of Pathogenic Biology, School of Basic Medical Sciences, Xinxiang Medical University, Xinxiang, China; ^2^Department of Dermatology, The First Affiliated Hospital of Xinxiang Medical University, Xinxiang, China; ^3^Department of Cardiology, The Third Affiliated Hospital of Xinxiang Medical University, Xinxiang, China; ^4^Department of Immunology, School of Basic Medical Sciences, Xinxiang Medical University, Xinxiang, China

**Keywords:** *Eucommia ulmoides*, gut microbiota, *Parabacteroides*, hypertension, IL-17A

## Abstract

Hypertension is a major threat to human health. *Eucommia ulmoides* Oliv. (EU) is a small tree and EU extract is widely used to improve hypertension in East Asia. However, its major constituents have poor absorption and stay in the gut for a long time. The role of the gut microbiota in the anti-hypertensive effects of EU is unclear. Here, we examined the anti-hypertensive effects of EU in high-salt diet and N(omega)-nitro-L-arginine methyl ester (L-NAME) induced mice. After receiving EU for 6 weeks, the blood pressure was significantly reduced and the kidney injury was improved. Additionally, EU restored the levels of inflammatory cytokines, such as serum interleukin (IL)-6 and IL-17A, and renal IL-17A. The diversity and composition of the gut microbiota were influenced by administration of EU; 40 significantly upregulated and 107 significantly downregulated amplicon sequence variants (ASVs) were identified after administration of EU. ASV403 (*Parabacteroides*) was selected as a potential anti-hypertensive ASV. Its closest strain XGB65 was isolated. Furthermore, animal studies confirmed that *Parabacteroides* strain XGB65 exerted anti-hypertensive effects, possibly by reducing levels of inflammatory cytokines, such as renal IL-17A. Our study is the first to report that EU reduces blood pressure by regulating the gut microbiota, and it enriches the *Parabacteroides* strain, which exerts anti-hypertensive effects. These findings provide directions for developing novel anti-hypertensive treatments by combining probiotics and prebiotics.

## Introduction

Hypertension is a major threat to human health, as it is a major risk factor for death from stroke, cardiovascular disease, and renal disease (Forouzanfar et al., 2017; Furie, 2020; Mills et al., 2020; Zhou et al., 2021a,b). In 1990, 648 million people suffered from hypertension, and this was increased to 1,278 million in 2019 (Zhou et al., 2021a). The pathogenesis of hypertension is complex, involving genetic, and environmental factors, such as high-salt diet (HSD), obesity, age, alcohol consumption, smoking, and stress (Vallianou et al., 2020). Most hypertensive patients undergo long-term treatment with medication that causes side effects, which should not be underestimated (Hu et al., 2020). Therefore, it is vital to develop new drugs to alleviate hypertension.

*Eucommia ulmoides* Oliv. (EU) is a small tree and EU extract is widely used in East Asia (Wu et al., 2018; Ding et al., 2020), due to its wide range of pharmacological effects such as anti-hypertension, anti-inflammatory, anti-hyperlipidemia, anti-diabetes, anti-oxidant, and anti-tumor effects (Shao et al., 2015; Zhang et al., 2022). In particular, the EU bark has been used for thousands of years in traditional Chinese medicine to improve hypertension (Li et al., 2020). The chemical constituents of the EU bark extract include lignans, iridoids, flavonoids, phenols, steroids, fatty acids, volatile oils, antifungal proteins, and gutta-percha (He et al., 2018; Wang C. Y. et al., 2019). Among them, lignans, flavonoids, and iridoids are the major anti-hypertensive constituents of EU bark extract. For example, lignans are reported to improve hypertension by regulating the nitric oxide level, renin–angiotensin system, and direct arterial diastole (Luo et al., 2010), inhibiting inflammatory and oxidative stress signaling pathways, promoting cardiovascular remodeling (Gu et al., 2011; Li et al., 2013), and ameliorating renal damage (Li et al., 2012) in spontaneously hypertensive rats (SHRs). Quercetin, a flavonoid extracted from EU, has an anti-hypertensive effect by improving vascular compliance and resistance, body fluid volume, the autonomic nervous system, and the renin–angiotensin system (Marunaka et al., 2017). Geniposidic acid, an iridoid extracted from EU, lowers blood pressure by inhibiting the expression of NADPH oxidase, upregulating endothelial nitric oxide synthase, and improving renal plasma flow (Ishimitsu et al., 2021). However, the major constituents of EU such as lignans, flavonoids, and iridoids, have poor oral bioavailability (Zhu et al., 2015; Ramakrishna et al., 2016; Eid and Haddad, 2017; Teodor et al., 2020), so it is unclear why EU has a good anti-hypertensive effect. Recent studies indicated that its effects on the gut microbiota might be involved.

Gut dysbiosis is reported in hypertensive animal models and patients, with reduced microbial diversity and a disordered structure (Yang et al., 2015; Li et al., 2017; Sharma et al., 2019; Sun et al., 2019). Further studies indicate that hypertension can be improved by altering the gut microbiota (Mell et al., 2015; Yang et al., 2015, 2018; Naqvi et al., 2021; Palmu et al., 2021; Xiong et al., 2021; Yano and Niiranen, 2021). Reduced blood pressure was observed in hypertensive animals receiving fecal microbiota transplantation (FMT) from normotensive animals (Toral et al., 2019a,b), which confirmed a causal relationship between the gut microbiota and hypertension. Studies identified possible bacteria involved in the regulation of blood pressure. For instance, *Lactobacillus* (Wilck et al., 2017; Zhang et al., 2019), *Roseburia* (Li et al., 2017; Yan et al., 2017; Calderón-Pérez et al., 2020; Silveira-Nunes et al., 2020; Stevens et al., 2021), *Coprococcus* (Li et al., 2017; Kim et al., 2020; Silveira-Nunes et al., 2020), *Akkermansia* (Sun et al., 2019; Kim et al., 2020; Zhou et al., 2021c), and *Bifidobacterium* (Li et al., 2017; Stevens et al., 2021) increase with decreasing blood pressure. However, the causal relationships between single strains and hypertension are poorly studied, except regarding the commonly used probiotics *Lactobacillus* and *Bifidobacterium* (Xu et al., 2013; Guilian et al., 2015; Guimarães et al., 2020; Robles-Vera et al., 2020a; de la Visitación et al., 2021; Machado et al., 2021). In this study, the aims were to clarify the anti-hypertensive effect of EU and the mechanism (potentially involving the gut microbiota) in a hypertensive mouse model involving an HSD and N(omega)-nitro-L-arginine methyl ester (L-NAME) treatment, and to identify a potentially beneficial bacterial strain that could be used for hypertension treatment.

## Materials and methods

### Preparation of *Eucommia ulmoides*

Using distilled water (2,000 mL each time), 0.4 kg of dried EU bark underwent extraction three times (20 min each time) at 100°C. Using an ALPHA 1–4 LDplus freeze-dryer (Martin Christ Gefriertrocknungsanlagen GmbH, Osterode am Harz, Germany), the crude extract was concentrated to a final volume of 20 mL. Thereafter, it was mixed with 5 kg of 4% high-salt feed (Xietong Co., Ltd., China), which was dried to reach a final concentration of 8% EU.

### Animals

Male specific-pathogen-free C57BL/6J mice (8 weeks old) were purchased from Beijing Vital River Laboratory Animal Technology Co., Ltd. (Beijing, China). The mice were maintained in individually ventilated caging systems under standard laboratory conditions with a 12-h light/dark cycle. The mice were provided regular chow and water *ad libitum*. For adaptation, they were housed for 1 week before the experiment. Animal care was performed according to the National Institutes of Health’s Guide for the Care and Use of Laboratory Animals and the Guidelines for the Ethical Review of Laboratory Animal Welfare (People’s Republic of China National Standard GB/T 35892-2018) (MacArthur Clark and Sun, 2020). The experimental protocol was approved by the Institutional Animal Care and Use Committee of Xinxiang Medical University.

### Mouse model of hypertension and treatment with *Eucommia ulmoides*

Six mice were fed a normal diet and drinking water as normal diets (ND) group (one of them were stopped from further experiment because of the abnormal blood pressure). An HSD- and L-NAME-induced mouse model of hypertension was established. First, the mice (*n* = 16) initially received 0.5 mg/mL of L-NAME (Meilunbio, Dalian, China) in the drinking water for 2 weeks, followed by a 1-week washout period. They were then fed a high-salt diet (4% NaCl; Xietong Co., Ltd., Nanjing, China) for 3 weeks. Thereafter, they were randomly divided into two groups that were fed the following diets for 6 weeks (from week 7 to the end of week 12): HSD group (4% NaCl; *n* = 6) and HSD + EU group (4% NaCl and 8% EU; *n* = 10). According to the principle of Reduction, Replacement, Refinement, and Responsibility, 6 mice were used in each group. To keep the results reliably after administration of EU and predict the potential anti-hypertensive bacteria more accurately, we increased the sample sizes in the EU + HSD group.

### Blood pressure measurement

After 7 days of pre-training regarding blood pressure measurement, the blood pressure of conscious mice was measured at the end of each week using a non-invasive computerized tail-cuff system (medlab, NJKEWBIO, China). Mean blood pressure values were determined based on at least seven successful measurements during the daytime.

### Collection of feces, blood, and kidney samples

Feces samples were collected in weeks 10 and 13. Blood samples were collected in week 13 and centrifuged at 4,000 × g for 5 min to obtain the serum. In week 13, both the left and right kidneys were excised from the mice. Feces, serum, and left kidneys were stored at −80°C, while the right kidneys were kept in 4% paraformaldehyde.

### Histomorphology examination

The right kidney samples were fixed in 4% paraformaldehyde for 24 h, embedded in paraffin, and cut into 3-μm-thick sections. Hematoxylin–eosin (HE) staining was performed to observe the histomorphology.

### Measurement of serum aldosterone, lipopolysaccharide, and inflammatory cytokines

Serum aldosterone and lipopolysaccharide (LPS) were measured using enzyme-linked immunosorbent assay (ELISA) kits (Jiancheng Co., Ltd., China). Serum inflammatory cytokines (interferon [IFN]-γ, tumor necrosis factor [TNF]-α, IL-10, IL-22, IL-6, and IL-17A) were measured using a LEGENDplex*™* MU Th17 Panel (7-plex) with a V-bottom Plate kit (BioLegend, United States) on a flow cytometer (CytoFLEX, Beckman, Inc.).

### Measurement of renal inflammatory cytokines and CYP11B2 using RT-qPCR

The left kidney samples were homogenized and the total RNA was extracted using RNAiso Plus (Takara, China) according to the manufacturer’s protocol. Isolated RNA was reverse transcribed using PrimeScript RT master mix (Takara, China). RT-qPCR was performed using SYBR Green PCR Core Reagent (Takara, China) in an ABI StepOne Plus Real-Time PCR system (Applied Biosystems, United States). The primer sequences for TNF-α, IL-6, IL-17A, and CYP11B2 (aldosterone synthase gene) are shown in Supplementary Table 1. The expression levels were normalized to glyceraldehyde 3-phosphate dehydrogenase (GAPDH) mRNA, and the relative expression was calculated using the −ΔΔCt method.

### DNA extraction, PCR amplification, and Illumina sequencing

Genomic DNA of the fecal samples was extracted using a Biomiga Stool gDNA Miniprep kit (Biomiga, United States) according to the manufacturer’s protocol. The V3–V4 region of the bacterial 16S rRNA gene was amplified by PCR (98°C for 1 min, followed by 30 cycles at 98°C for 10 s, 50°C for 30 s, 72°C for 30 s and a final extension at 72°C for 5 min) using primers 338F (5′-barcode-ACTCCTACGGGAGGCAGCA-3′) and 806R (5′-GGACTACHVGGGTWTCTAAT-3′) (Huws et al., 2007), where the barcode was a six-base sequence unique to each sample. PCR amplification was conducted using Phusion High-Fidelity PCR Master Mix (New England Biolabs, United States) and amplicons were purified using a Qiagen Gel Extraction Kit (Qiagen, Germany). Sequencing libraries were generated using an Illumina TruSeq DNA PCR-Free Library Preparation Kit (Illumina, United States) following the manufacturer’s protocol, and index codes were then added. The library quality was assessed on a Qubit 2.0 Fluorometer (Thermo Scientific, United States) and a 2100 Bioanalyzer System (Agilent Technologies, United States). Finally, the qualified libraries were sequenced on an Illumina NovaSeq platform belonging to Novogene Co., Ltd., (China), which generated 250-bp paired-end reads. The raw reads were deposited in the Sequence Read Archive of the National Center for Biotechnology Information (NCBI; accession number PRJNA846985).

### Processing of sequencing data

Using QIIME 2 (Bolyen et al., 2019), quality filtering and denoising of raw reads were conducted, and then the taxonomic classification of each 16S rRNA gene sequence was determined based on the SILVA (SSU 132) 16S rRNA database. Diversity was assessed based on the Shannon index, Faith’s phylogenetic diversity [PD; i.e., diversity based on a phylogenetic tree (Faith, 1992)], and Observed Features (i.e., richness of the bacterial community), which were calculated using QIIME 2 (Bolyen et al., 2019).

### Isolation of *Parabacteroides* strain XGB65

To confirm whether a *Parabacteroides* strain can regulate blood pressure, fecal samples from the mice in the EU + HSD group in week 13 were cultured to isolate *Parabacteroides* strains. A 100 μL sample of each 10^5^-diluted fecal suspension was spread-plated on modified Gifu anaerobic medium, brain-heart infusion medium, and chopped meat medium (Haibo, China). The plates were then incubated at 37°C in an anaerobic workstation (10% CO_2_, 10% H_2_, and 80% N_2_). Three replicates of each fecal sample were spread-plated in each medium. After 72 h of incubation, 20 colonies were randomly selected from each plate and purified. For DNA-based identification of the strains, the bacterial cultures were added to 20 μL sterile distilled water and stored at 4°C overnight. Next, 2 μL of this mixture was amplified with EasyTaq DNA Polymerase (Transgen, China) using the universal 16S rRNA gene primers 27F (5′-AGAGTTTGATCMTGGCTCAG-3′) and 1492R (5′-TACGGYTACCTTGTTACGACTT-3′). The purified PCR products were sequenced with an ABI PRISM automatic sequencer (model 3730XL, Sangon Biotech Co., Ltd., China). The 16S rRNA gene sequences were compared to the available 16S rRNA gene sequences in GenBank for identification, using BLAST and a web-based tool at http://www.ezbiocloud.net, as described by Yoon et al. (2017). We examined the differences in gut bacterial community composition between the EU + HSD and HSD groups at the amplicon sequence variant (ASV) level. A *Parabacteroides* strain (XGB65) with 98.34% identity to the V3-V4 region of ASV403 (which was selected because it was an ASV with a classified genus, > threefold change regarding EU + HSD group vs. HSD group, and *p* < 0.01) was isolated and stored at -80°C in 20% glycerol.

### Administration of *Parabacteroides* strain XGB65

An HSD- and L-NAME-induced mouse model of hypertension was established, as previously described in the “Mouse model of hypertension and treatment with EU” section. The mice received 0.5 mg/mL L-NAME (Meilunbio, Dalian, China) daily in drinking water for 2 weeks, followed by a 1-week washout period. To further clarify that whether *Parabacteroides* strain XGB65 can lower the blood pressure at the early stage of hypertension, the treatment was started from the 4th week. At the beginning of week 4, the mice were randomly divided into two groups (*n* = 6 per group) and treated as follows for 3 weeks (from week 4 to the end of week 6): NS + HSD group (HSD and 200 μL oral normal saline daily) and XGB65 + HSD group (HSD and 200 μL 10^9^ colony-forming units (CFU)/mL oral suspension of *Parabacteroides* strain XGB65 daily). Another 6 mice were fed a normal diet and drinking water throughout the period of experiment and received 200 μL oral normal saline daily from week 4 to the end of week 6 as NS + ND group.

Blood pressure was measured once a week. After treatment administration for 3 weeks, kidney samples were collected for HE staining and immunofluorescence staining. The kidney sections were incubated with antibody against interleukin (IL)-17A (1:500 dilution; Proteintech Group, Inc., China) overnight at 37°C for 1 h. Thereafter, the sections were incubated with goat anti-rabbit fluorescein isothiocyanate (FITC)-labeled antibody (1:500 dilution; Beyotime Biotechnology, China) for 30 min at room temperature. The samples were then washed five times with phosphate-buffered saline and observed under a fluorescence microscope at 200 × magnification. The mean fluorescence intensity was used to indicate IL-17A protein expression.

### Statistical analysis

Statistical analysis was performed using R software (version 4.1.2). Between-group comparisons of blood pressure, serum aldosterone, serum LPS, serum inflammatory cytokines (IFN-γ, TNF-α, IL-10, IL-22, IL-6, and IL-17A), renal relative mRNA expression [TNF-α, IL-6, IL-17A, and CYP11B2 (aldosterone synthase gene)], renal IL-17A protein expression (based on immunofluorescence), and diversity indices involved one-way analysis of variance (ANOVA) followed by least significant difference (LSD) tests. Between-groups comparisons of relative abundances of ASVs involved Wilcoxon rank-sum tests. A value of *p* < 0.05 was considered to be statistically significant. Principal co-ordinates analysis (PCoA) and permutational multivariate analysis of variance (PERMANOVA) were used to compare the gut bacterial community composition among the groups, using R software (version 4.1.2) with the vegan package (R Core Team, 2018). R software (version 4.1.2) was also used to create scatter, box, line, violin, bar, and heatmap plots (R Core Team, 2018).

## Results

### *Eucommia ulmoides* improved blood pressure and ameliorated kidney injury

**FIGURE 1:**
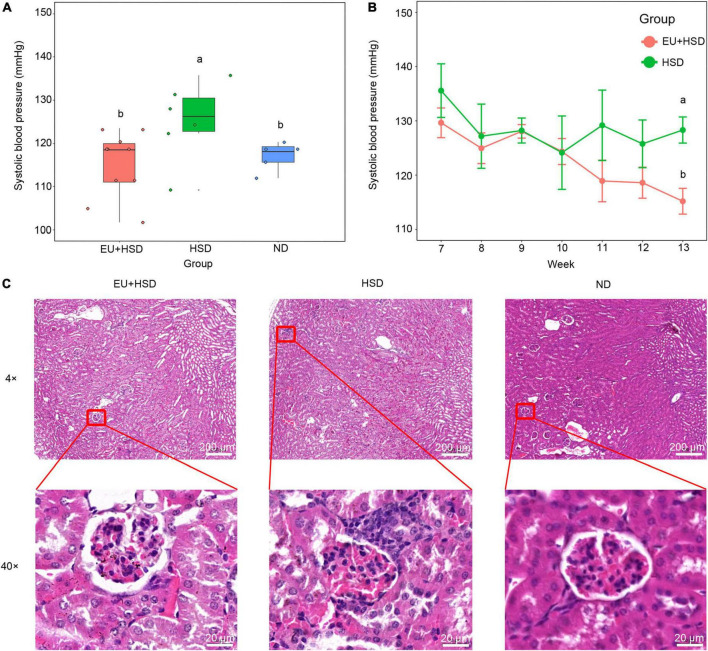
Anti-hypertensive effect of EU. Blood pressure **(A)** in the EU + HSD, HSD, and ND groups in week 13 and **(B)** in the EU + HSD and HSD groups from weeks 7 to 13. **(C)** HE staining of kidney tissues in the EU + HSD, HSD, and ND groups.

EU was administered to the EU + HSD group from week 7 to the end of week 12. Blood pressure in week 13 was significantly higher in the HSD group than the ND group, and significantly lower in the EU + HSD group than the HSD group (Figure 1A). In week 11, the blood pressure in the EU + HSD group started to be reduced compared to the HSD group, but no significant difference was observed (Figure 1B). According to the histopathological examination of the kidney tissues, compared to the ND group, the mice in the HSD group exhibited enlarged glomeruli, reduced size of Bowman space, and inflammatory infiltration, which were improved in the EU + HSD group (Figure 1C).

### *Eucommia ulmoides* restored serum IL-6 and IL-17A and renal IL-17A

**FIGURE 2:**
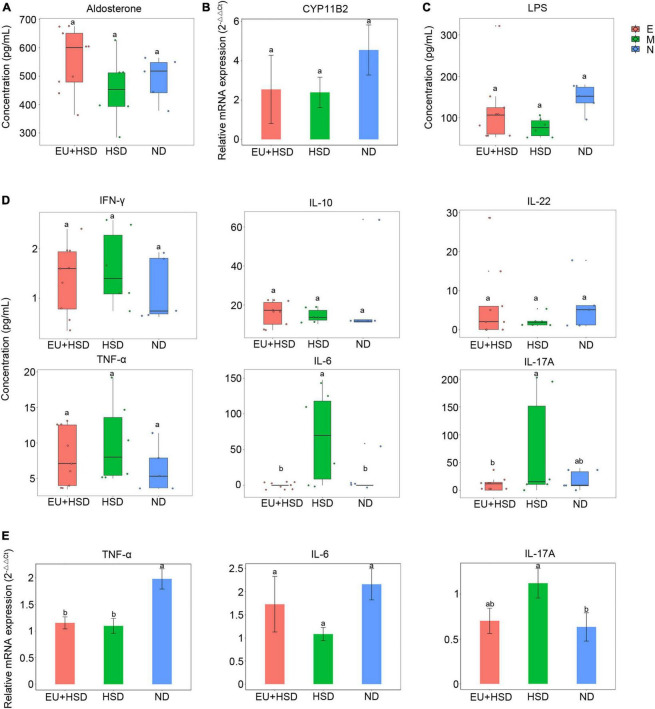
Aldosterone, CYP11B2, LPS, and inflammatory cytokine profiles in week 13 after administration of EU. **(A)** Serum aldosterone. **(B)** Relative expression of renal CYP11B2 mRNA. **(C)** Serum LPS. **(D)** Serum IFN-γ, IL-10, IL-22, TNF-α, IL-6, and IL-17A. **(E)** Relative expression of renal TNF-α, IL-6, and IL-17A mRNA. Values are shown as mean ± standard error of the mean (SEM).

To investigate the anti-inflammatory effects and hormonal regulation effects of EU, the serum aldosterone, serum LPS, serum inflammatory cytokines, and renal inflammatory cytokines and CYP11B2 (aldosterone synthase gene) were measured by ELISA, flow cytometry, or RT-qPCR. The serum aldosterone and renal CYP11B2 were not significantly changed among the groups (Figures 2A,B). The serum LPS (Figure 2C), IFN-γ, IL-10, and IL-22 (Figure 2D) were also not significantly changed. The serum IL-6 and IL-17A were significantly lower in the EU + HSD group than the HSD group, while the serum TNF-α was non-significantly lower (Figure 2D). We further confirmed the relative expressions of renal TNF-α, IL-6, and IL-17A mRNA (Figure 2E). Renal IL-17A was reduced in the EU + HSD group compared to the HSD group, whereas renal TNF-α and IL-6 were not significantly different.

### Regulation of gut microbiota by *Eucommia ulmoides*

**FIGURE 3:**
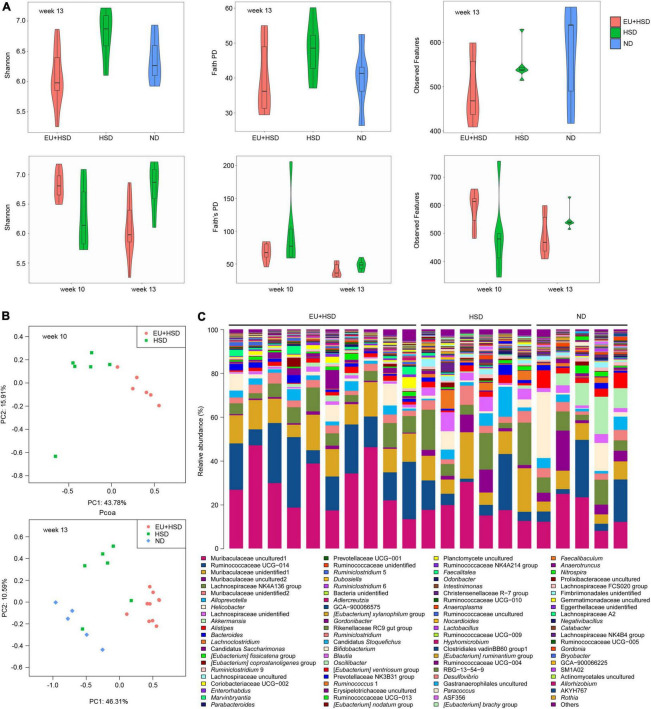
EU influences gut microbiota diversity and composition in hypertensive mice subjected to a high-salt diet (HSD) and L-NAME treatment. **(A)** Shannon index, Faith’s PD, and Observed Features in the EU + HSD, HSD, and ND groups in week 13 and the EU + HSD and HSD groups in weeks 10 and 13. **(B)** Principal co-ordinates analysis of bacterial communities in the EU + HSD and HSD groups in week 10 and in the EU + HSD, HSD, and ND groups in week 13. PC1: principal component 1; PC2: principal component 2. **(C)** Relative abundances of dominant genera (> 0.03%) in the EU + HSD, HSD, and ND groups in week 13.

The gut microbiota is involved in the regulation of blood pressure (Mell et al., 2015; Yang et al., 2015, 2018). The effect of EU on the diversity and composition of the gut microbiota was explored using high-throughput sequencing in weeks 10 and 13. A total of 1,566,542 valid reads and a mean of 47,471 reads per sample were obtained (Supplementary Table 2). Rarefaction curves based on the Shannon and Faith’s PD indices indicated that the dominant ASVs were captured (Supplementary Figure 1). In week 13, the highest Shannon and Faith’s PD indices were observed in the HSD group, while the lowest Shannon, Faith’s PD, and Observed Features indices were observed in the EU + HSD group (Figure 3A). One-way ANOVA demonstrated that there were significant differences in the Shannon indices between the EU + HSD and HSD groups in week 13 (*p* < 0.01). Furthermore, the diversity indices decreased from weeks 10 to 13 in the EU + HSD group. These results indicated that EU decreased the gut bacterial diversity.

PCoA and PERMANOVA showed that the composition of the gut bacterial community differed significantly among the groups in weeks 10 and 13 (Figure 3B and Supplementary Tables 3, 4). Surprisingly, blood pressure was not reduced by EU in week 10 (though it was significantly reduced in week 13), yet the composition of the gut bacterial community was changed significantly in week 10, which suggested that EU might reduce the blood pressure *via* the gut bacteria.

Bar plots show the dominant phyla and genera in week 13 (Figure 3C, Supplementary Figure 2, and Supplementary Tables 5, 6). The dominant phyla were Bacteroidetes (53.9%), Firmicutes (35.2%), Actinobacteria (3.3%), Epsilonbacteraeota (3.2%), and Verrucomicrobia (0.8%) in the EU + HSD group; Bacteroidetes (51.4%), Firmicutes (41.4%), Epsilonbacteraeota (2.7%), Actinobacteria (1.8%), and Patescibacteria (0.6%) in the HSD group; and Bacteroidetes (44.1%), Firmicutes (34.5%), Epsilonbacteraeota (9.2%), Verrucomicrobia (8.4%), and Actinobacteria (1.7%) in the ND group. The relative abundance of the phylum Firmicutes was increased while Actinobacteria, Epsilonbacteraeota, and Verrucomicrobia were decreased in the EU + HSD group compared to the HSD group (Supplementary Figure 2 and Supplementary Table 5).

The dominant genera were Muribaculaceae unidentified1, Ruminococcaceae UCG-014, Muribaculaceae unidentified2, Muribaculaceae unidentified3, and Lachnospiraceae NK4A136 group, which are unidentified or uncultured genera (Figure 3C and Supplementary Table 6). Therefore, to find a bacterial strain involved in the anti-hypertensive effect of EU, we further examined the differences in gut bacterial community composition between the EU + HSD and HSD groups at the ASV level.

### Amplicon sequence variants potentially involved in regulating blood pressure

**FIGURE 4:**
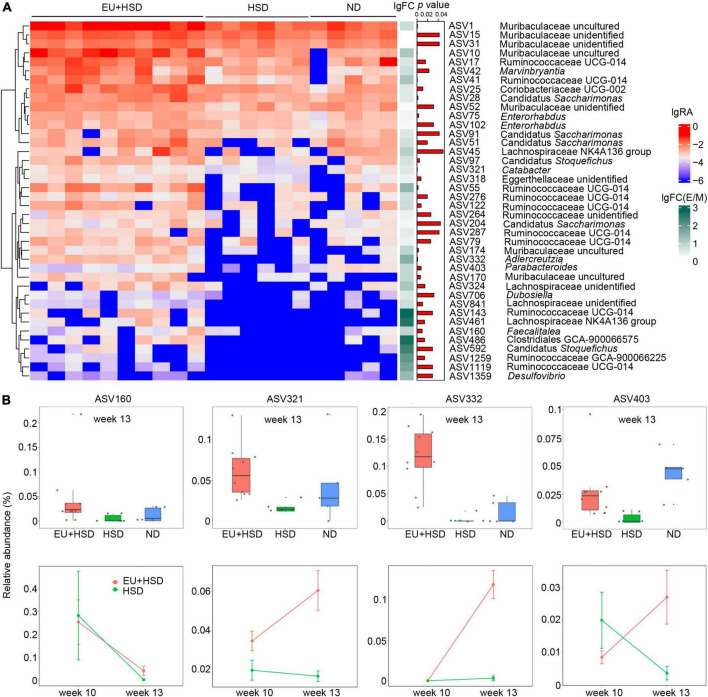
ASVs upregulated by EU treatment. **(A)** Heatmap indicating the significantly upregulated and downregulated ASVs in the EU + HSD group compared to the HSD group. **(B)** Relative abundances of four upregulated ASVs (*p* < 0.01, EU + HSD vs. HSD group > 3-fold difference) with classified genera in the EU + HSD, HSD, and ND groups in week 13 and the EU + HSD and HSD groups in weeks 10 and 13.

In the EU + HSD group compared to the HSD group, 40 ASVs were significantly upregulated and 107 ASVs were significantly downregulated (Figure 4A and Supplementary Figure 3). To find the ASVs with potential anti-hypertensive effects, the upregulated ASVs were further analyzed. ASV143 (Ruminococcaceae UCG-014), ASV486 (Lachnospiraceae GCA-900066575), ASV461 (Lachnospiraceae NK4A136 group), ASV592 (Candidatus Stoquefichus), and ASV1259 (Ruminococcaceae GCA-900066225) had the highest fold change regarding EU + HSD group vs. HSD group, but they are all unclassified genera, making them difficult to study further.

Therefore, we selected the ASVs with a classified genus, > 3-fold change regarding EU + HSD group vs. HSD group, and *p* < 0.01. These ASVs included ASV160 (*Faecalitalea*), ASV321 (*Catabacter*), ASV332 (*Adlercreutzia*), and ASV403 (*Parabacteroides*) (Figure 4B). In week 13, the four ASVs were all reduced in the HSD group compared to the ND group, but only ASV403 had a significant difference (*p* < 0.01) (Figure 4B), which suggested that its relative abundance was decreased in the HSD group and then restored by EU.

Furthermore, the changes of the four ASVs over time were observed (in weeks 10 and 13, Figure 4B). From week 10 to 13, the relative abundance of ASV160 was decreased in the EU + HSD and HSD groups. The relative abundances of ASV321 and ASV322 were increased in the EU + HSD group, while there were no obvious changes in the HSD group. Interestingly, the relative abundance of ASV403 was increased in the EU + HSD group and decreased in the HSD group (Figure 4B). ASV403 was selected to undergo assessment of its anti-hypertensive effect, to confirm that EU acts *via* its effect on gut bacteria.

### *Parabacteroides* strain XGB65 reduced blood pressure and renal IL-17A

**FIGURE 5:**
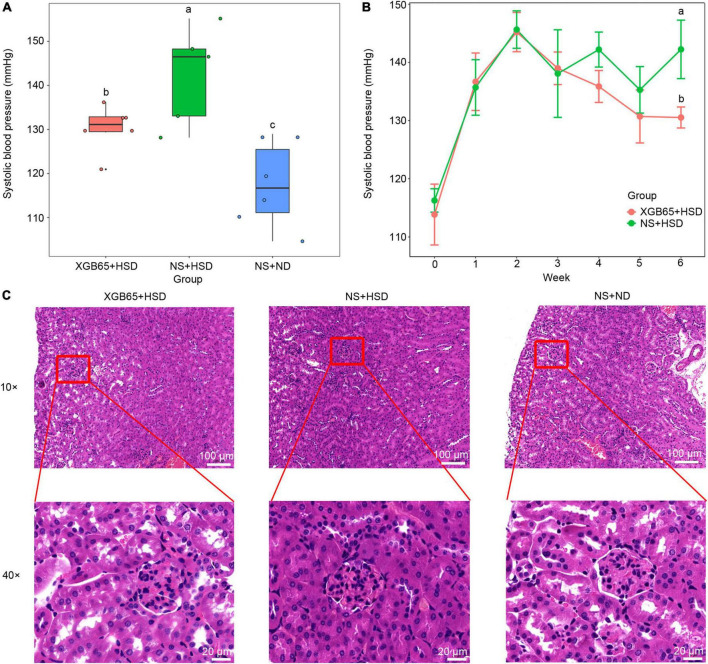
*Parabacteroides* strain XGB65 reduces blood pressure. Blood pressure **(A)** in the XGB65 + HSD, NS + HSD, and NS + ND groups in week 6 and **(B)** in the XGB65 + HSD and NS + HSD groups from the beginning of the experiment to the end of week 6 (2-week L-NAME treatment, 1-week washout period, XGB65 + HSD treatment from week 4 to week 6). **(C)** HE staining of kidney tissues in the XGB65 + HSD, NS + HSD, and NS + ND groups (10 ×, scale bars = 100 μm; 40 ×, scale bars = 20 μm).

The V3-V4 sequence of the 16S rRNA gene of *Parabacteroides* strain XGB65, which was isolated from feces in the EU + HSD group, had 98.4% similarity to ASV403. In the second set of animal experiments, *Parabacteroides* strain XGB65 was administered to the mice in the XGB65 + HSD group from week 4 to the end of week 6. At the end of the administration (at the end of week 6), blood pressure was significantly higher in the XGB65 + HSD and NS + HSD groups than the NS + ND group, while it was significantly reduced in the XGB65 + HSD group compared to the NS + HSD group (Figure 5A). At the end of week 4 (after XGB65 treatment had been administered for 1 week), the blood pressure in the XGB65 + HSD group started to non-significantly decrease compared to the NS + HSD group (Figure 5B).

Histopathological examination indicated that the kidneys in the NS + HSD group exhibited a slightly reduced size of Bowman space with no other obvious pathological changes compared to the NS + ND group, and there were no obvious improvements in the XGB65 + HSD group compared to the NS + HSD group (Figure 5C), which may have been due to the short treatment duration.

**FIGURE 6:**
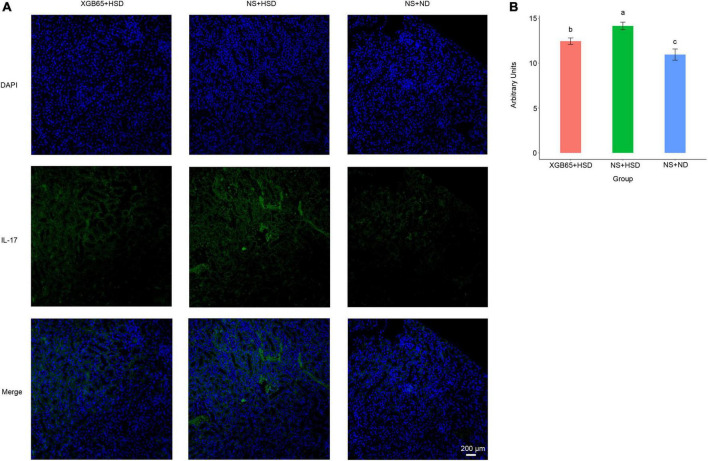
*Parabacteroides* strain XGB65 restores renal IL-17A. **(A)** IL-17A immunofluorescence in kidney sections in the XGB65 + HSD, NS + HSD, and NS + ND groups (scale bars = 200 μm). **(B)** Mean fluorescence intensity of IL-17A was significantly increased in the NS + HSD group compared to the NS + ND group, while it was significantly decreased in the XGB65 + HSD group compared to the NS + HSD group.

Furthermore, IL-17A protein expression was investigated using immunofluorescence. It was significantly increased in the NS + HSD group compared to the NS + ND group, and significantly decreased in the XGB65 + HSD group compared to the NS + HSD group (Figures 6A,B). These results indicated that *Parabacteroides* strain XGB65 reduced blood pressure and restored the renal IL-17A level.

## Discussion

Herbs always have extensive pharmacological activities, but their mechanisms are often complex and unclear. In particular, developing treatments involving herbs with low oral bioavailability is a challenge. The interactions between herbs and the gut microbiota may be a key mechanism of action for herb extracts with low oral bioavailability but excellent activities. For example, most active constituents of EU have low oral bioavailability (Zhu et al., 2015; Ramakrishna et al., 2016; Eid and Haddad, 2017; Teodor et al., 2020). This suggests that the gut microbiota may play an important role in the anti-hypertensive effects of EU. In the current study, we demonstrated that EU restored blood pressure, lowered inflammatory cytokines, and regulated the diversity and composition of the gut microbiota. Thereafter, ASV403 (*Parabacteroides*) was identified as a potential anti-hypertensive ASV. The closest strain, XGB65, was then isolated. Further animal studies confirmed that *Parabacteroides* strain XGB65 had an anti-hypertensive effect, possibly by reducing the inflammatory responses (such as by decreasing renal IL-17A). This study reveals the interactions among herbs, gut microbes, and inflammatory responses in hypertension, which provides a basis for developing new anti-hypertensive probiotics and prebiotics.

In this study, EU improved the blood pressure and ameliorated the kidney injury in hypertensive mice subjected to an HSD and L-NAME treatment, which is consistent with previous findings in Dahl salt-sensitive rats (Ishimitsu et al., 2021) and SHRs (Li et al., 2012, 2020). For example, *Eucommia* lignans ameliorated renal dysfunction, with decreased aldose reductase and collagen type III, in SHRs and inhibited the proliferation of renal mesangial cells induced by angiotensin II (Li et al., 2012). EU and its extract geniposidic acid also reduced blood pressure and improved renal hemodynamics in Dahl salt-sensitive hypertensive rats (Ishimitsu et al., 2021). Inflammation is involved in the development of hypertension (Barbaro et al., 2015; Zhang et al., 2022), while EU can reduce the inflammation. For instance, our study suggests that EU reduces serum IL-6 and IL-17A, and renal IL-17A, which is consistent with previous studies. Wang et al. (2016) and Xing et al. (2020) showed that EU inhibited the expression of splenic IL-6 and IL-17 mRNA and decreased serum IL-17 in collagen-induced arthritis rats. Wang J. Y. et al. (2019) also suggested that EU male flower extract suppressed IL-6 secretion and IL-17 mRNA expression in LPS-stimulated RAW 264.7 cells. As far as we know, this is the first evidence that EU lowers blood pressure, ameliorates kidney injury, and reduces inflammation in HSD and L-NAME induced mice. The good anti-hypertensive effect and poor oral bioavailability remind us that the gut microbiota may be involved in the anti-hypertensive effect of EU.

Administration of EU changed the diversity and composition of the gut microbiota. Although lower microbial diversity has been considered as a potential risk factor regarding hypertension in some research (Yano, 2020), other studies indicate that diversity is not a key indicator in a rat model of pulmonary arterial hypertension (Callejo et al., 2018) and a cohort of hypertensive patients (Mushtaq et al., 2019). In the current study, hypertensive mice had a higher microbial diversity than normal mice, and EU decreased the microbial diversity so that it became similar to that of normal mice. Therefore, we speculated that it might be beneficial to keep the diversity balance for improving hypertension. The decreased diversity after administration of EU may occur because gut microbes may be selectively inhibited/promoted by the constituents of EU. For instance, quercetin can selectively inhibit gut bacteria, having no inhibitory effect on *Ruminococcus gauvreauii*, a low inhibitory effect on *Lactobacillus* strains, and a mildly inhibitory effect on *Enterococcus caccae* (Firrman et al., 2016; Dos Santos et al., 2019). Moreover, lignans can stimulate the growth of *Lactobacillus* strains and inhibit the growth of *Enterococcus faecalis* (Favela-Hernandez et al., 2012; Stojanov et al., 2021). Increased *Lactobacillus* and decreased *Enterococcus* can help to lower blood pressure (Gómez-Guzmán et al., 2015; Wilck et al., 2017; Robles-Vera et al., 2020b; Zhu et al., 2021). In addition, in the current study, EU significantly changed the composition of the gut microbiota, which is consistent with a previous study on the anti-hypertensive effects of the combination of *Eucommia ulmoides* and *Tribulus terrestris* (Qi et al., 2020). In particular, the composition of the gut microbiota in our study was significantly changed in week 10 before the blood pressure was significantly decreased, which suggested that EU might reduce the blood pressure *via* the gut microbiota.

To reveal the potential gut bacteria involved in the anti-hypertensive effect of EU at the strain level, four ASVs (ASV160, ASV321, ASV332, and ASV403) were identified, which belong to *Faecalitalea*, *Catabacter*, *Adlercreutzia*, and *Parabacteroides*, respectively. However, emerging evidence indicates that these genera may be positively associated with hypertension. *Faecalitalea* was significantly increased in spontaneously hypertensive heart failure rats compared to both Wistar Kyoto rats and SHRs (Gutiérrez-Calabrés et al., 2020). *Christensenella* is a basonym for *Catabacter*, which was positively associated with hypertension in White and Black participants (Louca et al., 2021). *Christensenella timonensis* may serve as a biomarker in hypoxia-induced pulmonary hypertension mice (Luo et al., 2021). *Adlercreutzia* was increased in hypertensive heart failure rats compared to the normal group (Li et al., 2021). *Parabacteroides* was significantly enriched in mice with angiotensin II-induced hypertension mice (Sharma et al., 2019) and hypertensive patients. In contrast, other studies suggested that *Parabacteroides* was negatively correlated with hypertension. For example, *Parabacteroides* was associated with persistently lowered blood pressure in SHRs (Yang et al., 2019). *Parabacteroides gordonii* was also decreased in the rats which received FMT from HSD-induced hypertensive rats (Abais-Battad et al., 2021). These contradictory results may be due to different strains in the same genus having different (pro- or anti-hypertensive) effects.

Previous studies suggested that *Parabacteroides* could alleviate several diseases such as obesity and metabolic dysfunction (Wang K. et al., 2019), acute pancreatitis (Lei et al., 2021), multiple sclerosis, type II diabetes, colorectal cancer, and inflammatory bowel disease (Ezeji et al., 2021). Thus, *Parabacteroides* could potentially be developed into probiotics for human health. However, to our knowledge, there are no previous experiments on the anti-hypertensive effect of administering *Parabacteroides* strains. In this study, we identified *Parabacteroides* strain XGB65 (the closest strain to ASV403) and we further investigated its anti-hypertensive effect. The strain showed an excellent anti-hypertensive effect even in the first week after administration (though the effect was non-significant in the first week). *Parabacteroides* is one of several bacteria found in the colon with anti-inflammatory effects (Khan et al., 2018), as it decreases the TNF-α level in macrophages and IL-17 expression in colonic tissue (Kverka et al., 2011; Ishioka et al., 2017). These findings are consistent with the current finding that *Parabacteroides* strain XGB65 reduces the inflammatory cytokine IL-17A in the kidneys.

IL-17 may be a key factor in the anti-hypertensive effects of *Parabacteroides* strain XGB65. It is well known that an HSD is a major trigger of hypertension (O’Donnell et al., 2014; Mozaffarian et al., 2014), and the hypertensive mouse model involving an HSD and L-NAME treatment in this study is closely related to the salt-sensitive hypertension common in humans (Wilck et al., 2017). High salt increases the level of Th17 cells (Kleinewietfeld et al., 2013; Wu et al., 2013), which increases IL-17A release. IL-17A has numerous hypertensive effects, such as increasing salt retention, which contributes to blood volume increase (Norlander et al., 2016). Therefore, *Parabacteroides* strain XGB65 may reduce blood pressure by decreasing IL-17A, which is similar to the observation that *Lactobacillus murinus* administration prevents salt-sensitive hypertension by modulating Th17 cells (Wilck et al., 2017).

## Conclusion

Our study provides the first evidence that EU reduces blood pressure by regulating the gut microbiota. Administration of EU in HSD- and L-NAME induced hypertensive mice improved blood pressure and ameliorated kidney injury, lowered inflammatory cytokines (serum IL-6 and IL-17A, and renal IL-17A), and regulated the diversity and composition of the gut microbiota. Thus, ASVs significantly upregulated or downregulated by EU were identified. ASV403 (*Parabacteroides*) was selected as a potential anti-hypertensive ASV, and the closest strain (XGB65) was then isolated. Furthermore, animal studies confirmed that *Parabacteroides* strain XGB65 might exert an anti-hypertensive effect by reducing the inflammatory cytokine IL-17A in the kidneys. The relationships among the *Parabacteroides* strain, immunity, and hypertension require further study. As this study provides the first evidence that *Parabacteroides* exerts an anti-hypertensive effect, the results may provide a novel approach for developing anti-hypertensive treatments involving probiotics and prebiotics.

## Data availability statement

The raw data reads were deposited in the NCBI Sequence Read Archive database under accession number: PRJNA846985.

## Ethics statement

The animal experiments were conducted in accordance with the principles provided by the National Institutes of Health’s Guide for the Care and Use of Laboratory Animals and Use of Laboratory Animals and the Guidelines for the Ethical Review of Laboratory Animal Welfare (People’s Republic of China National Standard GB/T 35892–2018) (MacArthur Clark and Sun, 2020), and the experimental protocol was approved by the Institutional Animal Care and Use Committee of Xinxiang Medical University.

## Author contributions

XS, HS, and DY designed the study. MeY, YuC, YaC, and JL investigated the anti-hypertensive effects of EU in mice. WS, XZ, YL, KW, MoY, and FL investigated the anti-hypertensive effects of *Parabacteroides* strain XGB65 in mice. DY, WS, and XZ wrote the manuscript. DY, ML, XW, and MW analyzed the data. ZT, HS, and XS revised the manuscript. All authors reviewed and commented on the manuscript, contributed significantly to the preparation of the manuscript, and approved the submission of this manuscript.
