# Supplementary material for: Eucommia ulmoides bark extract reduces blood pressure and inflammation by regulating the gut microbiota and enriching the Parabacteroides strain in high-salt diet and N(omega)-nitro-L-arginine methyl ester induced mice
Source: Front Microbiol. 2022 Aug 18;13:967649. doi: 10.3389/fmicb.2022.967649 (PMC9434109; doi:10.3389/fmicb.2022.967649)
Supplement: Supplementary file 1 [file Data_Sheet_1.DOCX]

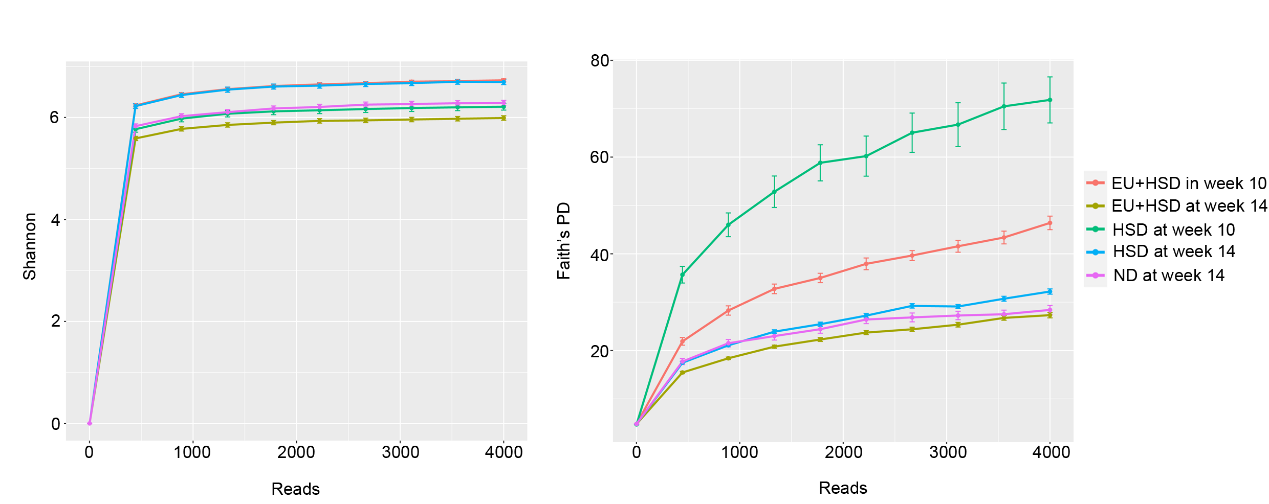


**Supplementary** **Figure S1**: **Rarefaction curves** **based on Shannon and Faith’s PD indices.**


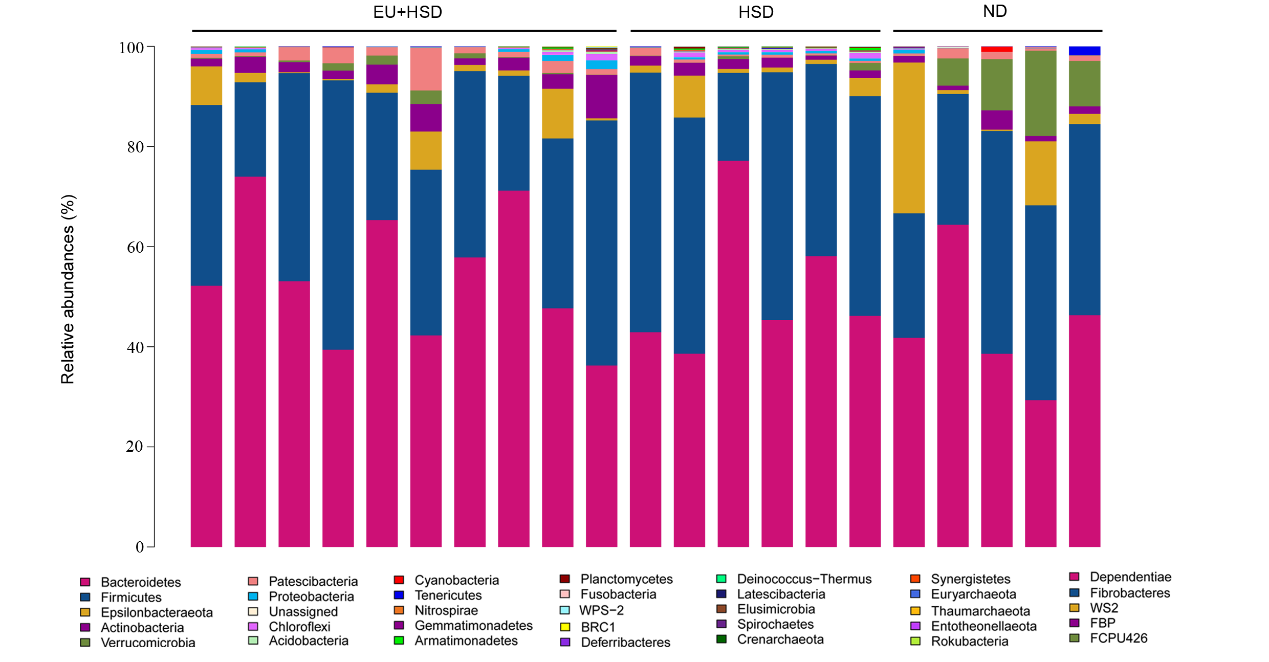


**Supplementary** **Figure S2**: **Bar plot indicating the relative abundances of phyla.**


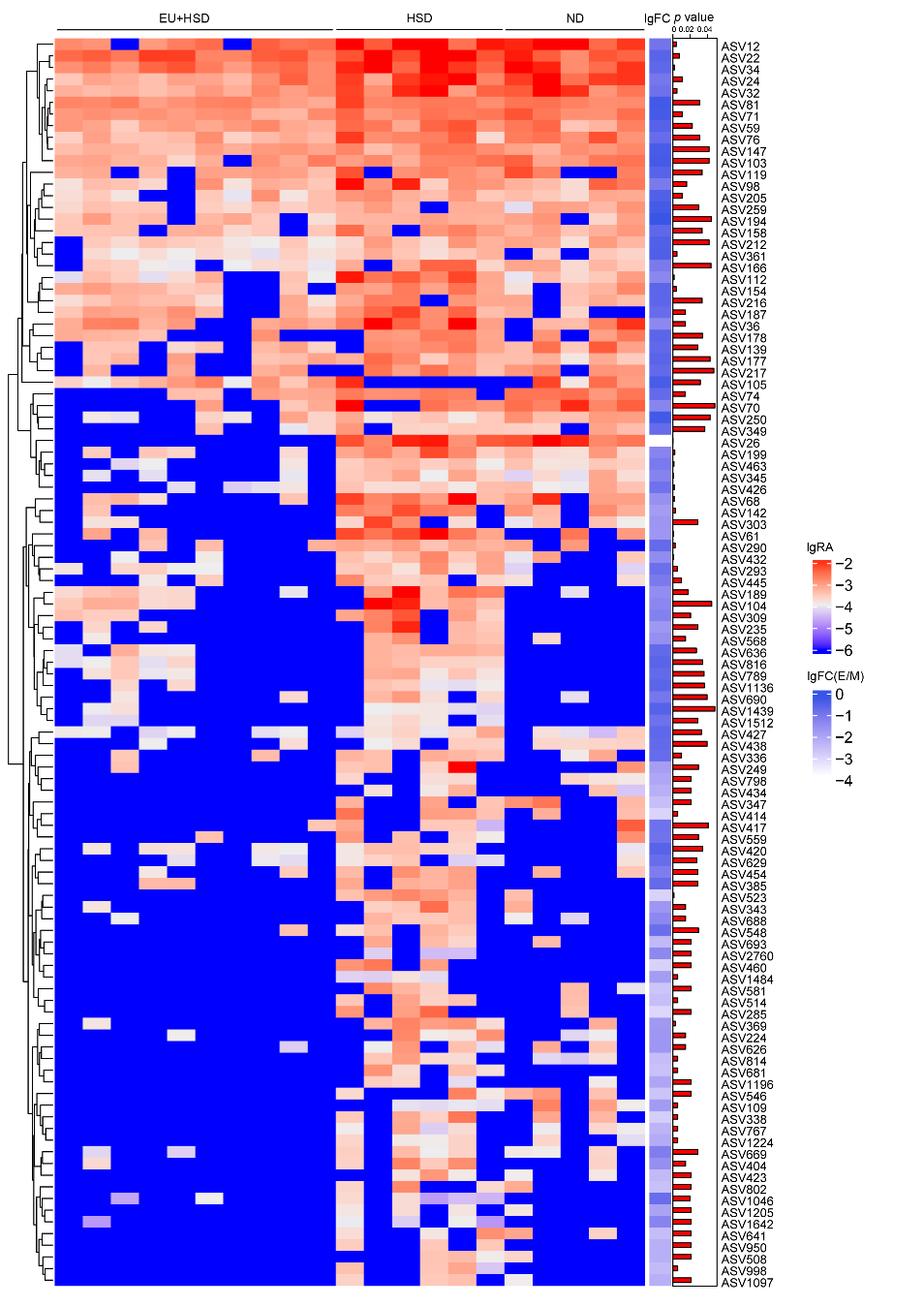


**Supplementary** **Figure S3**: **Heatmap indicating the significantly downregulated ASVs in the EU+HSD group compared to the HSD group.**
